# Isolation and Characterization of A2-EPTX-Nsm1a, a Secretory Phospholipase A_2_ from Malaysian Spitting Cobra (*Naja sumatrana*) Venom

**DOI:** 10.3390/toxins13120859

**Published:** 2021-12-02

**Authors:** Nur Atiqah Haizum Abdullah, Muhamad Rusdi Ahmad Rusmili, Syafiq Asnawi Zainal Abidin, Mohd Farooq Shaikh, Wayne C. Hodgson, Iekhsan Othman

**Affiliations:** 1 Jeffrey Cheah School of Medicine and Health Sciences, Monash University Malaysia, Jalan Lagoon Selatan, Bandar Sunway, Subang Jaya 47500, Malaysia; syafiq.asnawi@monash.edu (S.A.Z.A.); farooq.shaikh@monash.edu (M.F.S.); 2 Centre for Tissue Engineering and Regenerative Medicine, Faculty of Medicine, Universiti Kebangsaan Malaysia, Jalan Yaacob Latif, Bandar Tun Razak, Kuala Lumpur 56000, Malaysia; 3 Kulliyyah of Pharmacy, Kuantan Campus, International Islamic University Malaysia, Bandar Indera Mahkota, Kuantan 25200, Malaysia; rusdirusmili@iium.edu.my; 4 Monash Venom Group, Department of Pharmacology, Biomedical Discovery Institute, Monash University, Clayton, VIC 3800, Australia; Wayne.Hodgson@monash.edu

**Keywords:** *Naja sumatrana*, spitting cobra, snake venom phospholipase, phospholipase A_2_

## Abstract

Phospholipase A_2_ (PLA_2_) toxins are one of the main toxin families found in snake venom. PLA_2_ toxins are associated with various detrimental effects, including neurotoxicity, myotoxicity, hemostatic disturbances, nephrotoxicity, edema, and inflammation. Although *Naja sumatrana* venom contains substantial quantities of PLA_2_ components_,_ there is limited information on the function and activities of PLA_2_ toxins from the venom. In this study, a secretory PLA_2_ from the venom of Malaysian *N. sumatrana*, subsequently named A2-EPTX-Nsm1a, was isolated, purified, and characterized. A2-EPTX-Nsm1a was purified using a mass spectrometry-guided approach and multiple chromatography steps. Based on LC-MSMS, A2-EPTX-Nsm1a was found to show high sequence similarity with PLA_2_ from venoms of other *Naja* species. The PLA_2_ activity of A2-EPTX-Nsm1 was inhibited by 4-BPB and EDTA. A2-EPTX-Nsm1a was significantly less cytotoxic in a neuroblastoma cell line (SH-SY5Y) compared to crude venom and did not show a concentration-dependent cytotoxic activity. To our knowledge, this is the first study that characterizes and investigates the cytotoxicity of an Asp49 PLA_2_ isolated from Malaysian *N. sumatrana* venom in a human neuroblastoma cell line.

## 1. Introduction

*Naja sumatrana* (Sumatran cobra or Sunda spitting cobra) is a diurnal spitting cobra endemic in Malaysia, Singapore, Southern Thailand, Indonesia (Sumatra and Kalimantan), and the Philippines (Palawan and Calamianes Archipelago) [1,2]. *N. sumatrana* is considered to be a medically important species in the countries where it is found [3]. Systemic envenoming caused by *N. sumatrana* results in generalized paralysis, leading to death in the absence of proper clinical management and antivenom administration. Envenoming can also cause prominent localized dermonecrotic damage, causing morbidity due to disfigurement [4].

A venomic study of Malaysian *N. sumatrana* venom showed that the venom consists mainly of three-finger toxins and phospholipase A_2_ (PLA_2_) components [5]. Information on the activity of PLA_2_ toxins from *N. sumatrana* venom is lacking despite these components being one of the main constituents of the venom. Indeed, PLA_2_s are ubiquitous components in many snake venoms. Snake venom PLA_2_s are classified as secretory PLA_2_s and are divided into Group I and Group II based on their molecular weight, calcium-dependency, and catalytic residues [6]. Commonly, position 49 in the primary structure is composed of aspartic acid. However, in some species, this amino acid residue is replaced by lysine, arginine, asparagine, serine, or, rarely, cysteine. Modification at this position alters the enzymatic activity of the PLA_2_ by modifying its Ca^2+^ binding dependency [7] and contributes to causing a different type of toxicity [8].

PLA_2_ toxins isolated from elapid and viperid snake venoms have been reported to cause muscle necrosis [8,9,10,11,12,13,14,15,16], induction of inflammatory cytokines [9,12,15,17,18,19,20,21], neurotoxicity [15,22,23,24,25], edema [9,14,18,19,21], hypotension [26], vasoconstriction [27], hemolysis [28], pulmonary congestion [12], intraperitoneal bleeding [12], and acute kidney injury [29]. Some snake venom PLA_2_s have been reported to have potential therapeutic activities including anti-cancer [30,31,32,33,34,35], anti-angiogenic [36], antibacterial [37,38], anti-parasite [39], antithrombotic [40], anticoagulant [8,12,28,40,41], antiviral [42], neuronal survival [43], and platelet inhibition [44]. The mechanism of these activities depends on the targeted cells [45].

*N. sputatrix* was the previous taxonomical classification for the spitting cobra in the Malaysia–Singapore region [5]. However, only four sequences of PLA_2_ have been deposited in UniProt under the species name *N. sputatrix* [46,47,48]. Three isoforms of the PLA_2_ gene belonging to the acidic and neutral PLA_2_ gene (described NAJPLA-2A, NAJPLA-2B, and NAJPLA-2C) in *N. sputatrix* venom have been previously reported [46].

In this study, we have isolated, purified, and characterized a PLA_2_, that we named A2-EPTX-Nsm1a, from Malaysian *N. sumatrana* venom. The cytotoxicity of the PLA_2_ was determined using a neuroblastoma cell line. Information from this study will enhance the current knowledge on PLA_2_ in *N. sumatrana* and their activities.

## 2. Results

### 2.1. Purification of A2-EPTX-Nsm1a

**Figure 1:**
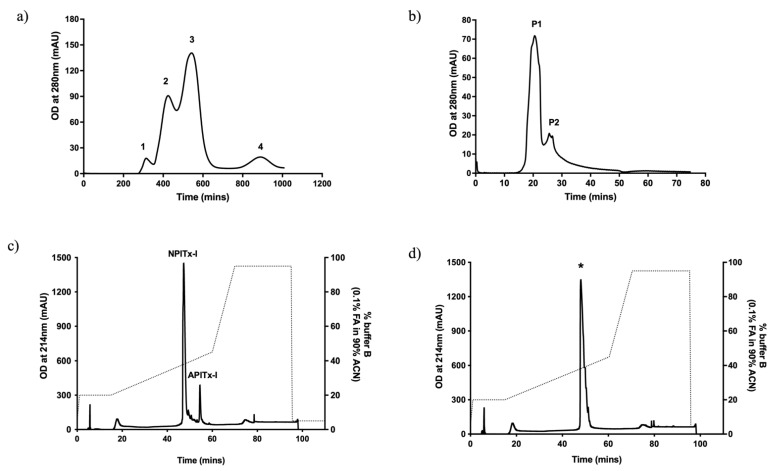
Sequential fractionation of A2-EPTX-Nsm1a from Malaysian *Naja sumatrana* venom. (**a**) Proteins with sequence similarity with PLA_2_ were identified in fraction 2 of Sephadex™ G50 by ESI-LC-MSMS. (**b**) Further fractionation using Superdex™ G75 separated the PLA_2_ from other proteins with similar protein mass and yielded two peaks (P1 and P2). (**c**) P1 was further fractionated with a reverse-phase column and separated into two PLA_2_ fractions (NPITx-I and APITx-I). (**d**) Isolated A2-EPTX-Nsm1a was detected in the peak indicated by * following additional reverse-phase chromatography.

A2-EPTX-Nsm1a was purified using a mass spectrometry-guided approach and sequential fractionation steps using gel filtration and reverse-phase chromatography. Crude venom separation using Sephadex™ G50 resulted in four fractions with fraction 2 containing a protein with a high degree of sequence similarity with PLA_2_ using ESI-LC-MS/MS analysis (Figure 1a). Further fractionation of fraction 2 using Superdex™ G75 yielded two distinct peaks (P1 and P2, Figure 1b) with peak 1 containing PLA_2_ based on ES-LC-MS/MS analysis. Peak 1 was further separated using reverse-phase chromatography and yielded two distinctive peaks labelled NPlTx-I and APlTx-II (Figure 1c, Supplementary data). Additional reverse-phase chromatography of NPlTx-I showed the presence of a single peak named A2-EPTX-Nsm1a based on the proposed nomenclature [49,50] (Figure 1d). The yield of A2-EPTX-Nsm1a at the end of the chromatographic steps was 7.1% based on the PLA_2_ specific activity.

### 2.2. SDS-PAGE

**Figure 2:**
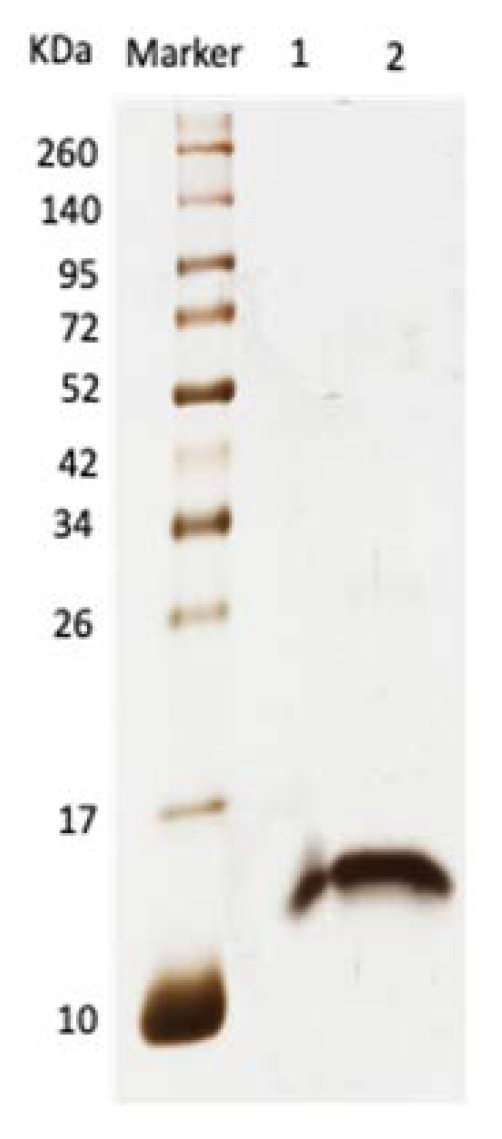
Silver-stained SDS-PAGE (10% acrylamide gel) lanes loaded with marker and A2-EPTX-Nsm1a under non-reduced (lane 1) and reduced (lane 2) conditions. The MW of A2-EPTX-Nsm1a estimated from SDS-PAGE is 13.6 KDa.

SDS-PAGE analysis for A2-EPTX-Nsm1a indicated a molecular weight of approximately 13–15 kDa under reduced and non-reduced conditions (Figure 2). Only a single band was seen for lanes loaded with A2-EPTX-Nsm1a (Figure 2).

### 2.3. Intact Protein Using Accurate Mass LC-MS

**Figure 3:**
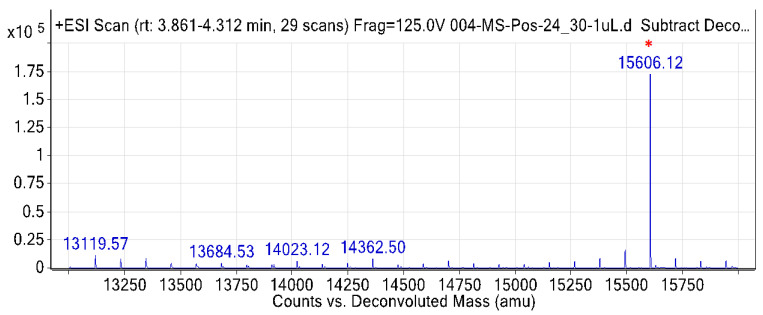
Intact protein mass analysis of *Naja sumatrana* A2-EPTX-Nsm1a by Agilent 6520 Accurate-Mass Q-TOF mass spectrometer. A dominant peak (*) indicated the molecular mass of A2-EPTX-Nsm1a.

Intact protein analysis using accurate mass LC-MS showed A2-EPTX-Nsm1a has a molecular weight of 15,606.12 Da (Figure 3). The tested sample showed the absence of other dominant proteins from 13,100–15,700 Da (Figure 3).

### 2.4. Protein Identification by ESI-LC-MSMS

ESI-LC-MSMS analysis on an excised SDS-PAGE band of A2-EPTX-Nsm1a revealed its sequence similarity with other PLA_2_ from *Naja* species (Table 1). The amino acid sequence was determined using SPIDER mode in PEAKS Studio X+ as below. Amino acids highlighted in grey and underlined are computationally detected in SPIDER mode and de novo sequencing, respectively.

1                                     50

**NLYQFKNMI QCTVPNRSWW HFADYGCYCG RGGSGTPVDD LDRCCQIHDNC**

51                                   100

**YNEAEKISR PYFK TYSYEC SQGTLTCKGG NNACAAAVCD CDRLAAICFAG**

101         115

**APYNDNNYN IDLKAR**

**Figure 4:**
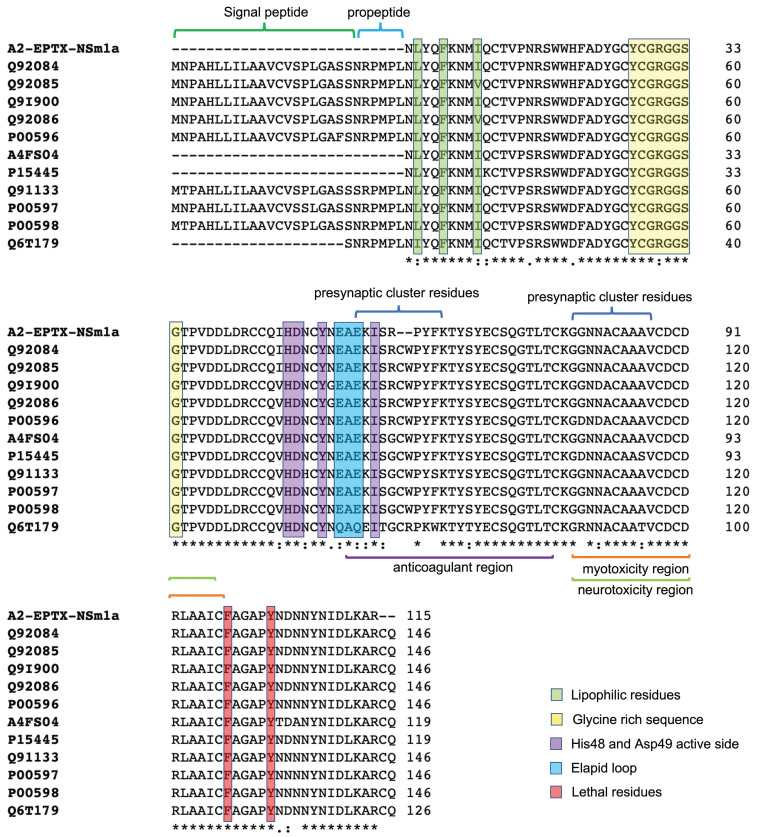
Alignment of A2-EPTX-Nsm1a amino acid sequence with amino acid sequence of matched proteins from LC-MSMS analysis using Clustal Omega Multiple Alignment. Identified important regions and residues are shown in this diagram. * indicates identical amino acid present in A2-EPTX-Nsm1a and matched proteins.

Multiple sequence alignment using the Clustal Omega program showed that the de novo sequence of A2-EPTX-NSm1a showed 76% sequence coverage with a neutral PLA_2_ muscarinic inhibitor from *Naja sputatrix* venom. The sequence also showed similarity with neutral PLA_2_ B from *Naja sputatrix* venom and other acidic phospholipase A_2_ from *N. sputatrix*, *N. kaouthia*, *N. naja*, *N. atra*, and *N. sagittifera* venoms (Table 1 and Figure 4).

Further analysis of the A2-EPTX-NSm1a peptide on its sequence function was performed using PROSITE (https://prosite.expasy.org accessed on 21 April 2021) to verify its catalytic site. A2-EPTX-NSm1 peptide at position 44–51 (CCQIHDNC) indicated a PLA_2_ histidine active site. Meanwhile, the A2-EPTX-NSm1a peptide at position 88–98 (VCDCDRLAAIC) is associated with the PLA_2_ aspartic acid active site. The estimated isoelectric point (pI) of A2-EPTX-Nsm1a obtained from Uniprot is 6.07.

### 2.5. Molecular Modelling

**Figure 5:**
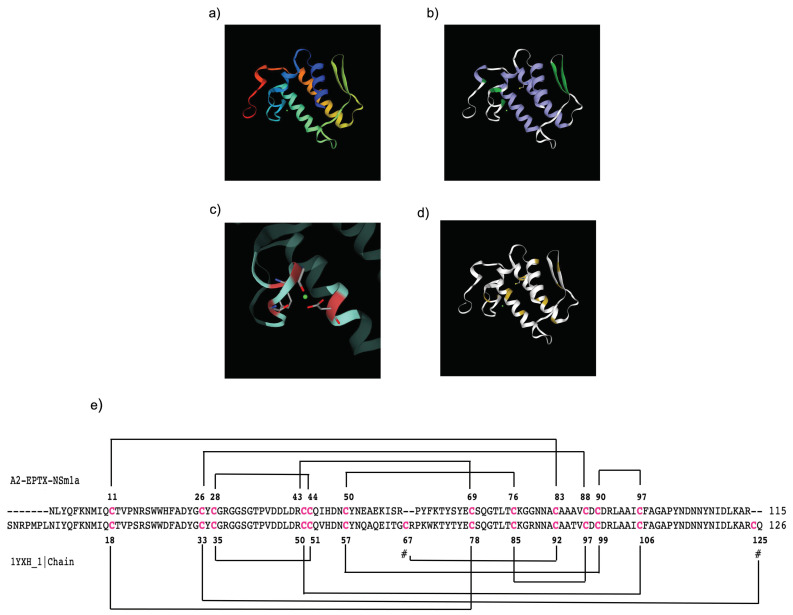
The theoretical structure of A2-EPTX-Nsm1a generated from SwissModel using SMTL ID: 1yxh.1, PLA_2_ from *Naja naja sagittifera* venom as the template (**a**). Homology molecular modelling cartoon indicates its secondary structure of α-helix and β-sheet structures (**b**) and the Ca^2+^ ligand (**c**). Cysteine residues present in the amino acid sequence of A2-EPTX-Nsm1a are highlighted in yellow (**d**). Prediction of disulfide link for A2-EPTX-Nsm1a using DiANNA 1.1 webserver showed 6 disulfide bridges and different disulfide link formations with the SMTL ID: 1yxh.1 template (**e**). These differences showed different interactions between the cysteine residues due to the absence of cysteine residues at the middle and C-terminal of the A2-EPTX-Nsm1a sequence (marked as #) (**e**).

The molecular modelling structure using the Swiss Model (https://swissmodel.expasy.org accessed on 21 April 2021) showed A2-EPTX-Nsm1a sequence matched with PLA_2_ from *Naja naja saggitifera* venom (SMTL ID: 1yxh.1). A rainbow-colored cartoon showed the N-terminal sequence (start with blue) to C-terminal sequence (end with red) (Figure 5a). The secondary structures of α-helix (colored with purple) and β-sheet (colored with green) and Ca^2+^ binding loops were determined in this 3D model (Figure 5b,c). The 12 cysteine residues along the sequence interacted to form six disulphide bridges proposed in this model (Figure 5d,e).

### 2.6. PLA_2_ Activity

**Figure 6:**
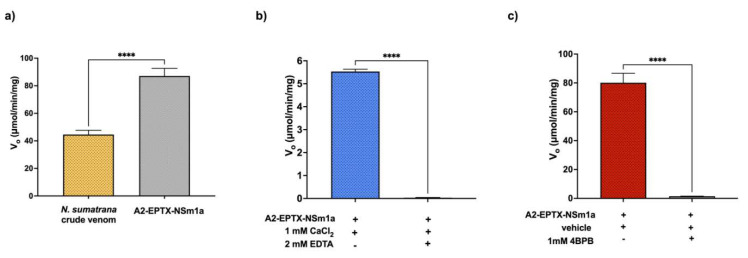
Secretory PLA_2_ activity of A2-EPTX-Nsm1a and the crude venom of *Naja sumatrana*. A2-EPTX-Nsm1a activity in the (**a**) absence of chemical modification and in (**b**) the presence of 2 mM EDTA or (**c**) 1.8 mM 4BPB. Acetone, which was used to dissolve 4BPB, was used as a negative control (i.e., vehicle) in (**c**). The experiment was performed in triplicate. Data were statistically analyzed using *t*-test; **** indicated *p* < 0.001.

Venom and all PLA_2_-rich fractions showed PLA_2_ activity except APlTx-I, which showed low PLA_2_ activity (Supplementary Figure S3). However, the PLA_2_ activity of the venom and PLA_2_-rich fractions were lower than in the bee-venom-positive control (461.7 ± 44.2 µmol/min/mg) (Supplementary Figure S3). A2-EPTX-Nsm1a has higher PLA_2_ activity (87.1 ± 5.6 µmol/min/mg) compared to the crude venom (44.6 ± 3.1 µmol/min/mg) (Figure 6a). His48 modification using 4-BPB and EDTA significantly reduced PLA_2_ activity of A2-EPTX-Nsm1a compared to the native protein (Figure 6b,c).

### 2.7. Cytotoxicity Activity of A2-EPTX-NSm1a on SH-SY5Y

**Figure 7:**
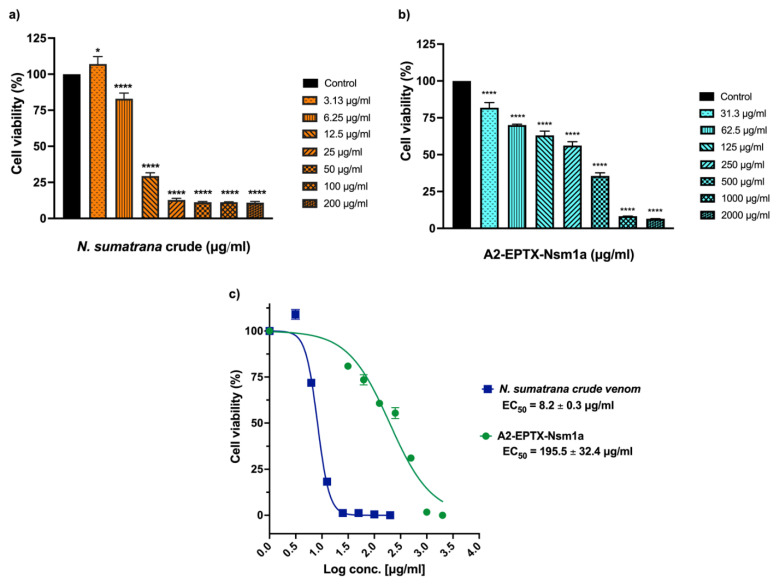
A2-EPTX-Nsm1a cytotoxicity against the neuroblastoma cell line, SH-SY5Y. (**a**) Concentration response of *N. sumatrana* crude venom and (**b**) secretory PLA_2_ isolated from *N. sumatrana* named A2-EPTX-Nsm1a. Cells were incubated with different concentrations of *N. sumatarana* crude venom and A2-EPTX-NSm1a for 24 h. MTT assay was performed to obtain the EC_50_ (concentration capable of reducing 50% of cell viability) curve (**c**) using Graph Pad Prism software. Cells without venom and A2-EPTX-NSm1a were used as control. Results were expressed as mean percentages (with control, considered 100%) ± SD (*n* = 5). * indicated *p* < 0.05, **** indicated *p* < 0.0001 compared to control.

The cytotoxic effects of A2-EPTX-NSm1a in the neuroblastoma cell line SH-SY5Y were determined using different protein concentrations. The EC_50_ was determined from a plotted graph then estimated based on the data. The EC_50_s of A2-EPTX-NSm1a and *N. sumatrana* crude venom were 195.5 ± 32.4 µg/mL and 8.2 ± 0.3 µg/mL, respectively. The results showed that A2-EPTX-NSm1a was less toxic to SH-SY5Y than its crude venom (Figure 7).

## 3. Discussion

Previous work has shown that the protein composition of *N. sumatrana* venom is relatively less complex than the composition of *N. kaouthia* venom [51,52]. The unique composition of spitting cobra venom, compared to the venom of non-spitting species, has likely evolved due to the defensive role of the venom in the former [53]. PLA_2_ toxins are highly expressed in spitting cobra venom and have been shown to activate sensory pain in neurons as a protective mechanism against aggressors [53]. In this work, we have isolated a PLA_2_ named A2-EPTX-Nsm1a, from Malaysian *N. sumatrana* venom, using a mass spectrometry-guided approach and chromatography techniques. Size exclusion and reverse-phase chromatography used in this study have been previously used to isolate various types of proteins from snake venom [9,54]. Multiple chromatography steps are usually necessary to isolate a particular toxin from venom due to the complexity of the venom composition [55,56,57]. To the best of our knowledge, the activity of the isolated proteins from *N. sumatrana* venom in neuronal brain cells has never been reported. Therefore, cytotoxicity of A2-EPTX-Nsm1a in the SH-SY5Y neuroblastoma brain cell line was determined.

Electrophoresed A2-EPTX-Nsm1a in reduced and non-reduced conditions showed a single band in the silver-stained SDS-PAGE gel. The gel indicates that A2-EPTX-Nsm1a is a monomeric PLA_2_. It was further confirmed, by intact protein analysis using LC-MSMS, that the determined mass of the 115 amino acid sequence of A2-EPTX-Nsm1a was 15,606 Da. These features are consistent with previously reported monomeric snake venom secretory PLA_2_ [58,59] that consist of 115–125 residues, with a lower molecular weight ~15KDa compared to other PLA_2_ classes [58,59,60]. Close examination of the predicted structure of A2-EPTX-NSm1a showed a similar structural arrangement with other snake venom PLA_2_. (Figure 5). Alignment of the A2-EPTX-Nsm1a sequence, obtained from ESI- LC-MSMS analysis, with other snake venom proteins in the UniProt database showed sequence similarity with different secretory PLA_2_ isolated from *N. sputatrix, N. kaouthia, N. atra, N. naja,* and *N. sagitiferra* venoms (Table 1). The primary sequence of A2-EPTX-Nsm1a has 76% sequence coverage with the neutral PLA_2_ muscarinic inhibitor (Q92084) (Table 1). These findings indicate that the molecular features and function of A2-EPTX-Nsm1a could be identical to the neutral phospholipase PLA_2_ muscarinic inhibitor.

PLA_2_ belongs to a large protein superfamily that differ in their amino acid sequences and positions of disulfide bonds. Secretory PLA_2_ derived from cobra venom are classified as group I PLAs. This group shares distinct structural characteristics of three long α-helices, two β-strands, and a Ca^2+^ binding loop [61]. Similarly, molecular modelling using a homologous PLA_2_ template (SMTL ID: 1yxh.1) showed identical structural characteristics (Figure 5a–c.). Although the molecular modelling predicted that A2-EPTX-Nsm1a has seven disulfide bridges, only 12 cysteine residues were detected in the primary sequence of A2-EPTX-NSm1a by LC-MSMS analysis (Figure 5d,e). This finding confirmed the characteristic of secreted PLA_2_ as globular cysteine-rich proteins with 6 to 8 disulfide bonds that ensure enzyme stability and resistance to proteolysis and denaturation. The presence of active sites is essential in PLA_2_ catalytic action, with this activity dependent on calcium ions as a cofactor [62,63]. Function prediction using PROSITE indicates that A2-EPTX-Nsm1a is an Asp49 PLA2, a type of PLA_2_ commonly found in snake venoms [10,27,64]. This aspartic active site is vital for the snake venom PLA_2_ catalytic network with His48, Tyr52, and Tyr64 residues [65]. The N-terminal amino acid residues of lipophilic residues, namely Leu2, Phe5, and Ile9, are highly conservative substrate regions with a hydrophobic site [66,67]. These amino acids were also found in the sequence of A2-EPTX-Nsm1a. The primary structure of A2-EPTX-Nsm1a showed the presence of a Ca^2+^ binding loop that accommodates a glycine-rich sequence (Tyr24-Gly25-Cys26-Tyr27-Cys28-Gly29-Arg30-Gly31-Gly32-Ser33-Gly34). The formation of a Ca^2+^ binding loop with Tyr28, Gly30, Gly32, and Asp49 in its secondary structure generated in SWISS-MODEL indicated its dependency on Ca^2+^ to stabilize the catalytic conformation (Figure 5d).

PLA_2_ isolated from cobra venom shares a similar structure with pancreatic PLA_2_ [61]. Thus, these PLA_2_ are classified as Group I PLA_2_, and are further divided into Group IA and IB. The insertion at positions 54–56, called elapid loop residues, which link α-helices and β-sheets, differentiates between Group IA and IB [61]. These elapid loop residues of Glu-Ala-Glu were identified in the A2-EPTX-Nsm1a sequence, suggesting that A2-EPTX-Nsm1a should be grouped in Group 1A with other cobra and krait PLA_2_. Unlike other PLA_2_ in the database, the primary sequence of A2-EPTX-NSm1a obtained using LC-MSMS does not have signal and pro-peptides (Figure 4). Therefore, N-terminal peptide sequencing using Edman degradation is required to confirm the complete amino acid for future work. The PLA_2_ activity of *N. sumatrana* venom has been determined in a previous study [68] and was also measured in this study (Figure 6a). A2-EPTX-Nsm1a was found to have approximately two times higher PLA_2_ activity compared to *N. sumatrana* venom (Figure 6a). Coincubation of A2-EPTX-Nsm1a with the chelating agent EDTA attenuated PLA_2_ activity (Figure 6b). Snake venom PLA_2_ is a Ca^2+^-dependent enzyme, and inhibition by EDTA would be reversed by restoration of the Ca^2+^ concentration [69]. This finding demonstrated the importance of metal ions for PLA_2_ action. Modification at His48 by bromophenylation of A2-EPTX-Nsm1a, using 4-BPB, also abolished PLA_2_ activity, indicating the importance of His-48 in the PLA_2_ activity of A2-EPTX-Nsm1a [70,71]. Various studies on neurotoxic and myotoxic PLA_2_ toxins have shown that His-48 played an essential role in PLA_2_ activity [71,72].

The neuroblastoma cell line SH-SY5Y has been used widely in neurobiology studies [73,74]. Cultures of SH-SY5Y contain two morphologically distinct phenotypes: Neuroblast-like cells and epithelial-like cells from its parental SK-N-SH cells [74,75]. N-type in SH-SY5Y has been reported to display characteristics of catecholaminergic neurons, such as the expression of tyrosine hydroxylase and dopamine-β-hydroxylase [74,75]. SK-N-SH cells also provide advantages in maintenance and cost compared to primary neurons and their human-derived cell line. In addition, the used of SH-SY5Y allows neurobiological studies on specific human proteins, which are not available in cell lines from other origins [74]. Even though SH-SY5Y can differentiate to mature neuron cells, both undifferentiated and differentiated SH-SY5Y have been utilized in cell culture models that require neuron-like cells [73,75,76,77,78,79,80,81]. In this study, A2-EPTX-Nsm1a demonstrated cytotoxic activity in undifferentiated SH-SY5Y. However, the cytotoxic effect of A2-EPTX-Nsm1a was less potent compared to *N. sumatrana* crude venom (Figure 7). This finding suggests A2-EPTX-Nsm1a is less toxic to the undifferentiated SH-SY5Y. Significant cytotoxic effects from *N. sumatrana* crude venom may be due to the presence of other toxins such as cytotoxins, cardiotoxins, and other types of PLA_2_ [5], which may have synergistic or potentiating effects [13]. Based on the findings from past studies, the enzymatic activity of PLA_2_ may contribute to the cytotoxic effect [82,83]. It has been reported that the Asp49 variant PLA_2_s are less toxic at the cellular level when compared with PLA_2_s with Lys49 [13,45]. The current study only used one type of cell line, and A2-EPTX-Nsm1a activity towards other cell lines is unknown. The role of His-48 and the calcium ion in the cytotoxic, neurotoxic, and myotoxic activities of the toxin were also unable to be determined due to the limited amount of purified toxin.

## 4. Conclusions

In conclusion, we have isolated and characterized A2-EPTX-Nsm1a, a secretory PLA_2_ from Malaysian *N. sumatrana* venom. To our knowledge, this is the first study reporting the cytotoxicity of secretory PLA_2_ from *N. sumatrana* venom on human neuron-like cells, SH-SY5Y. Further work is required to determine the mechanism of toxicity and its relationship with PLA_2_ activity using different types of cell lines and other in vitro methods. The study provided additional information on the effects of snake venom PLA_2_ in a neuronal cell line.

## 5. Materials and Methods

### 5.1. Chemicals

Ammonium acetate, acrylamide, N, N’-Methylenebisacrylamide, Tris-HCl, silver nitrate, and 4-bromophenacyl bromide were purchased from MERCK, Kenilworth, NJ, USA 1,4-Dithiothreitol, iodoacetamide, trifluoroacetic acid, formic acid, and thiazolyl blue tetrazolium bromide were purchased from SIGMA-Aldrich. EDTA was purchased from Promega, Madison, WI, USA, USA and Pierce™ Trypsin Protease MS Grade was purchased from Thermo Fischer Scientific, NY, New York USA. Dulbecco Modified Essential Medium (DMEM), High Glucose, fetal bovine serum, and antibiotic-antimycotic used for the cell culture were purchased from Gibco, New York, NY, USA.

### 5.2. Crude Venom

Pooled crude venom of *N. sumatrana* (*n* = 5) was obtained from Perlis, Northwest of Peninsular Malaysia. Snakes were milked using a sterile container covered with parafilm. The venom was transported on ice and immediately frozen at −80 °C when it arrived at Monash University Malaysia and freeze-dried using a FreeZone Benchtop Freeze Dry System (Labconco, KS, USA). Freeze-dried venom was weighed, labelled, and stored at −20 °C before use. When required, snake venom was dissolved in Milli-Q water unless stated otherwise. The research permit for venom collection was obtained from the Department of Wildlife and National Parks Peninsular Malaysia, Ministry of Energy and Natural Resources (HQ-00067-15-70).

### 5.3. Purification of A2-EPTX-Nsm1a

First, 30 mg of crude venom was dissolved in Milli-Q water and centrifuged at 1000 rpm for 5 min. Supernatants were collected, filtered using a syringe filter, and loaded into a C 16/100 column (GE Healthcare Life Sciences, Uppsala, Sweden) packed with Sephadex™ G-50 Fine (GE Healthcare Life Sciences, Uppsala, Sweden). The column was mounted onto the Äkta Prime Plus System (GE Healthcare Life Sciences, Uppsala, Sweden). The column was equilibrated with 0.1 M ammonium acetate, pH 6.8, and run at a 0.2 mL/min flow rate. The elution was monitored at 280 nm and collected automatically with 2 mL/tube. The fractions containing A2-EPTX-Nsm1a were pooled and freeze-dried. Then, 10 mg of the fraction was loaded into a Superdex™ 75 10/300 GL column (GE Healthcare Life Science, Uppsala, Sweden) and mounted onto the Äkta Purifier system (GE Healthcare Life Sciences, Uppsala, Sweden). The column was equilibrated with 0.1 M ammonium acetate, pH 6.8, and run at a flow rate of 0.1 mL/min, and the elution was monitored at 280 nm. The elution was collected automatically with 1 mL/tube by the system. The fraction containing the toxin from the second step was loaded on a Jupiter^®^ C18 reverse-phase column (Phenomenex, CA, USA) mounted on an Agilent 1260 high-pressure liquid chromatography (HPLC) system (Agilent Technologies, Santa Clara, CA, USA). The column was equilibrated with 5% acetonitrile (ACN) with 0.1% trifluoracetic acid (TFA) in water and run at a flow rate of 0.5 mL/min. The elution was monitored at 214 nm. The toxin was eluted with an increasing percentage of 90% ACN in 0.1% TFA in water using the following gradient: 5% for 5 min, 5–20% over 15 min, 20–40% for 40 min, 40–95% for 10 min, and continued for 30 min and 100–5% for 20 min. The peaks were automatically collected.

### 5.4. Protein Quantification by Bicinchoninic Acid (BCA) Assay

The protein concentration from every fractionation and purification process was measured using the Pierce™ BCA Protein Assay Kit (Thermo Fischer Scientific, IL, USA) as the manufacturer’s manual instructed. In brief, the sample (25 μL) or standard (25 μL) was loaded onto a 96-well plate in triplicate before 200 μL of the reagent buffer mix was added to each well. The plate was incubated at 37 °C for 30 min and then read at 562 nm using an EON™ microplate spectrophotometer (BioTek Instruments, VT, USA). The protein concentrations of the venom and fractions were estimated from the protein concentration standard curve.

### 5.5. Sodium Dodecyl Sulphate-Polyacrylamide Gel Electrophoresis (SDS-PAGE)

SDS-PAGE was performed as described in the previous method [84]. Briefly, 5 μg of the sample was treated with reducing and non-reducing sample buffers and loaded in separate wells using 10% glycine-acrylamide gel. The Spectra Multicolor Broad-Range Protein Ladder (Thermo Fischer Scientific, IL, USA) was used as the molecular weight marker. Protein bands were separated at 60V for 30 min and 120V for about 1.5 h using the Hoefer SE260 system (Hoefer Inc, MA, USA). The gel was then stained using silver staining, and the image was captured using the GE Image Scanner III Labscan 6.0 (GE Healthcare Life Sciences, Uppsala, Sweden).

### 5.6. Intact Protein Analysis with Electrospray-Ionisation Coupled with Mass-Spectrometry

The protein was loaded onto an Agilent Zorbax Eclipse XDB-C18 chip column mounted on the Agilent 1290 Infinity LC system coupled to the Agilent 6520 Accurate-Mass Q-TOF mass spectrometer with a dual ESI source (Agilent Technologies, Santa Clara, CA, USA). The chip column was run at 0.5 mL/min using 0.1% formic acid in water (solution A) and 0.1% formic acid in acetonitrile (solution B). The chip was equilibrated with 5% solution B, and the gradient used during the run was 5–100% solution B for 5 to 20 min and maintained with 100% buffer B for another 5 min. The polarity of the Q-TOF was set at positive, the capillary voltage at 4000 V, the fragmentor voltage at 125 V, the drying gas flow at 10 L/min, and a gas temperature of 300 °C. The intact protein spectrum was analyzed in MS-only mode with a range of 100–3200 m/z. The spectrum was deconvoluted using Agilent MassHunter Qualitative Analysis B.07.00 (Agilent Technologies, Santa Clara, CA, USA).

### 5.7. In-Gel Tryptic Digestion

The gel band of interest in SDS-PAGE was cut carefully and placed in the Lo-Bind Eppendorf tube. Ammonium bicarbonate (ABC; 200 mM) in 40% of ACN was added to the tube and incubated at 37 °C for 30 min. The supernatant was later discarded, and 200 µL of 10 mM DTT reducing buffer was added to the tube and incubated for 1 h at 56 °C. Later, the reduction buffer was removed, and 55 mM of iodoacetamide alkylation buffer was added, and incubation was performed in the dark for 30 min. The alkylation buffer was removed, and the gel band was washed with 50 mM ABC followed by 50 mM ABC in 50% ACN at room temperature. The gel was washed three times in ACN for 15 min at 37 °C. The gel piece was briefly centrifuged, and all liquid was discarded before trypsin was added for digestion at 37 °C overnight. The supernatant containing the digested peptide was transferred to a new Lo-Bind Eppendorf tube and labelled collection tube. The digestion peptide collection continued with 5% formic acid (FA), followed by 50% ACN in 5% FA and ACN. Gel bands were incubated at 37 °C for 15 min, and all supernatant was transferred in the same collection tube. Recovered peptides were dried using a vacuum concentrator and stored at −80 °C before mass spectrometry analysis.

### 5.8. Protein Identification with Tandem Mass Spectrometry (ESI-LCMS/MS)

The in-gel digested sample was analyzed using the Agilent 1200 HPLC-Chip/MS Interface, coupled with Agilent 6550 iFunnel Q-TOF LC/MS. The digested peptides were loaded onto an Agilent C18 300 Å Large Capacity Chip (Agilent Technologies, Santa Clara, CA, USA). The column was equilibrated with 0.1% formic acid in water (solution A). Peptides were eluted with an increasing gradient of 90% acetonitrile (ACN) in 0.1% formic acid (solution B) by the following gradient: 5–75% solution B from 0 to 30 min and 75% solution B from 39 to 47 min. The polarity of the Q-TOF was set at positive, the capillary voltage at 1800 V, the fragmentor voltage at 360 V, the drying gas flow at 11 L/min, and a gas temperature of 280 °C. The spectrum was obtained from Agilent MassHunter Qualitative Analysis B.07.00 (Agilent Technologies, Santa Clara, CA, USA).

### 5.9. Automated De Novo Sequencing

Protein identification by automated de novo sequencing was conducted using PEAKS Studio X+ (version 10.0 Plus, Bioinformatics Solution, Waterloo, ON, Canada). The homology search was performed by comparing the de novo sequence tag with the SwissProt Serpentes database from September 2017. In PEAKS Studio X+, SPIDER mode was used. The setting for the false detection rate (FDR) is 0.1%, and a -log p score more than 20 for protein identification was accepted. Matched protein identification was accepted for protein coverage above 50%.

### 5.10. Molecular Modelling

The protein sequence obtained from SPIDER Mode from Peaks Studio X+ was used to identify structure homology using 3D structure tools in SwissModel. The template with similar coverage to A2-EPTX-Nsm1a and a high QMGE score (~1.0) based on its oligo state were selected for model building. A qualified model based on its QMEAN (Z score < 1) was finalized, and its PDB format was used to identify the disulphide bridge using Disulphide by Design 2.0 [85]. The structure was confirmed using other tools such as trRosetta and Phyton 3.7, as previously described. The disulfide bonds were confirmed using Dianna 1.1 [86] and CYSPRED [87].

### 5.11. PLA_2_ Activity

PLA_2_ activity of each fraction and A2-EPTX-Nsm1a was confirmed using the secretory PLA_2_ assay kit according to the manufacturer’s protocol (Catalogue No: 765001, Cayman Chemical, MI, USA). The final concentration of 0.45 μg/mL fractions was tested in the assay with a 1.66 mM substrate. Bee venom was used as the positive control. The activity of PLA_2_ was monitored for 30 min, and the absorbance was recorded every 3 min at 414 nm at 25 °C. The activity of PLA_2_ was calculated based on the manufacturer’s protocol.

### 5.12. PLA_2_ Inhibition by 4-Bromophenacyl Bromide (4-BPB) and EDTA

4-BPB was dissolved in acetone and mixed with 4.5 μg/mL of the A2-EPTX-Nsm1a sample to produce a final concentration of 1.8 mM 4-BPB, as in the previously described method [83]. The EDTA inhibition assay was incubated with 2 mM EDTA for 16 h at 25 °C. In this assay, the assay buffer was diluted, containing 1.66 mM substrate, 1 mM calcium chloride, 1 mM potassium chloride, and 0.03 mM Triton-X. Both conditions were measured at 414 nm at 25 °C for 30 min using an EON™ microplate spectrophotometer (BioTek Instruments, VT, USA). PLA_2_ activity of modified and treated A2-EPTX-Nsm1a was determined and compared with the enzyme without an inhibitor using a secretory PLA_2_ assay kit (Cayman Chemical, MI, USA).

### 5.13. Cell Culture

The human neuroblastoma cell line SH-SY5Y (ATCC CRL-2266) was seeded at a density of 20,000 cells/cm^2^ in T75 flasks. Cells were cryopreserved below passage 15 to avoid senescence. The culture was maintained in Dulbecco Modified Essential Medium (DMEM) High Glucose (Gibco 10569-010) supplemented with 10% heat-inactivated fetal bovine serum (FBS) (Gibco 10270) and 1X Antibiotic-Antimycotic (Gibco 15240-062) in humidified 5% CO_2_ and 37 °C incubators. The culture medium was replaced every two days until the culture reached confluency (70–80%) for sub-culturing or differentiation.

### 5.14. Cytotoxicity of A2-EPTX-Nsm1a on SH-SY5Y cells

Cytotoxicity of A2-EPTX-Nsm1a on the undifferentiated neuroblastoma cell line, SH-SY5Y, was assessed using thiazolyl blue tetrazolium bromide (Sigma-Aldrich Corp., St. Louis, MO, USA) in an MTT assay. Cells were seeded with a density of 10,000 cells/cm^2^ in 96 wells and maintained in growth media as described above until it reached 70–80% confluency. Different concentrations of A2-EPTX-Nsm1a and crude venom were added to each well and incubated for 24 h at 37 °C in a 5% CO_2_ humidified incubator. At the end of treatment, the MTT reagent was added until its final concentration was 0.05 mg/mL in each well. The cells were further incubated for 4 h at 37 °C and in a 5% CO_2_ humidified atmosphere. The insoluble formazan, which resulted from oxidation of the added MTT to vital cells, was dissolved by adding DMSO, and the absorbance of formazan was determined using an EON™ microplate spectrophotometer (BioTek Instruments, Winooski, Vermont, USA) at 570nm. The relative viability of the cells was defined as the ratio of optical density of formazan produced by cells treated with A2-EPTX-Nsm1a to the optical density produced by control cells. For each treatment, the optical density of control cells was considered as 100% of viable cells.

### 5.15. Statistical Analysis

Student’s *t*-test and a one-way ANOVA evaluated the statistical analysis for comparing two and three groups, respectively. Each experiment was conducted in at least three replicates, and the results were reported as the means ± standard deviations (SD). Differences were considered significant if *p* < 0.05. 

## Figures and Tables

**Table 1:** Sequence similarity of A2-EPTX-Nsm1a with other proteins from Serpentes database using Peaks Studio X+. Identity of A2-EPTX-Nsm1a identified in UniProt.

Accession	−10lgP	Coverage (%)	No. of Peptides	Average Mass	Description	Origin	Identity (%)
Q92084	233.5	76	17	16,189	Neutral phospholipase A_2_ muscarinic inhibitor	*Naja sputatrix*	97.8
Q92085	224.51	72	15	16,175	Neutral phospholipase A_2_ B	*Naja sputatrix*	97.4
Q9I900	200.54	74	12	16,097	Acidic phospholipase A_2_ D	*Naja sputatrix*	94.9
Q92086	198.43	70	11	16,082	Acidic phospholipase A_2_ C	*Naja sputatrix*	94.9
P00596	184.86	64	10	16,271	Acidic phospholipase A_2_ 1	*Naja kaouthia*	94
P00598	179.51	63	8	16,013	Acidic phospholipase A_2_ 1	*Naja atra*	94
P00597	179.51	63	8	16,016	Acidic phospholipase A_2_ 2	*Naja kaouthia*	94
Q91133	179.00	54	7	15,949	Acidic phospholipase A_2_ 2	*Naja atra*	92.3
P15445	172.39	65	7	13,346	Acidic phospholipase A_2_ 2	*Naja naja*	92.3
A4FS04	161.13	77	7	13,188	Acidic phospholipase A_2_ natratoxin	*Naja atra*	92.3
Q6T179	149.66	51	6	14,198	Acidic phospholipase A_2_ 4 (fragment)	*Naja sagittifera*	87.2

## Data Availability

The data presented in this study are available in this article and supplementary materials.

## Supplementary Materials

The following are available online at https://www.mdpi.com/article/10.3390/toxins13120859/s1, Figure S1: SDS-PAGE of protein bands from the fraction eluted in the G75 Superdex™ column named P1 and P2 (Figure 1b). Figure S2: Proteins bands of PLA_2_ fractions (APlTx-I and NPlTx-I) and APlTx-I accurate mass. Figure S3: PLA2 activity of all fractions. Table S1a: Protein profile of fraction P1 eluted from G75 Superdex™ column. Table S1b: Protein profile of fraction P2 eluted from G75 Superdex™ column. Table S2a: Protein profile of fraction NPlTx-I eluted from C18 reverse-phase column. Table S2b: Protein profile of fraction APlTx-I eluted from C18 reverse-phase column.
