# Supplementary material for: Isolation and Characterization of A2-EPTX-Nsm1a, a Secretory Phospholipase A2 from Malaysian Spitting Cobra (Naja sumatrana) Venom
Source: Toxins (Basel). 2021 Dec 2;13(12):859. doi: 10.3390/toxins13120859 (PMC8709200; doi:10.3390/toxins13120859)
Supplement: Supplementary file 1 [file toxins-13-00859-s001.zip › toxins-1443446-supplementary.pdf]

toxins

MDPI

Supplementary data

Supplementary 1

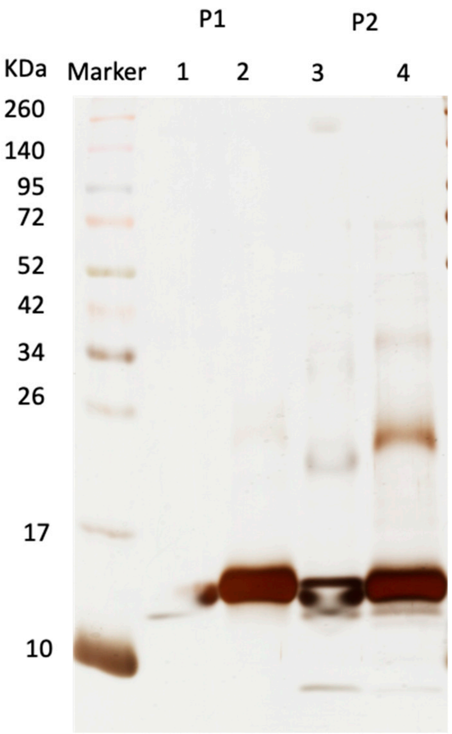

Supplementary Figure S1: SDS-PAGE of protein bands from the fraction eluted in the G75 Superdex™ column named P1 and P2 (Figure 1b). Five µg of protein bands in SDS-PAGE gel stained with silver staining. The first fraction eluted from the G75 Superdex™ column named P1 exhibited dominant protein bands in between the molecular weight of 15 to 26 KDalton in both reducing and nonreducing conditions (lanes 1 and 2). The second elution named P2 (lines 3 and 4) showed protein bands at broader size, up to 72 KDa. P2 protein bands revealed two bands at MW 20KDa and 15KDa at nonreducing condition (lane 3), respectively. Meanwhile, protein bands for P2 fractions exhibited four bands with estimated MW were 72KDa, 40KDa, 23KDa and 15KDa. All bands in each fraction (P1 & P2) were cut and digested with trypsin before subjected to ESI-LC-MSMS for its protein identification. All LC-MSMS data obtained in the fractions were pooled and presented in supplementary tables 1a and 1b.

Supplemental Table S1a: Protein profile of fraction P1 eluted from G75 Superdex™ column. Protein gel bands were tryptic digested and identified by ESI-LC-MSMS. Most of the proteins detected in P1 protein bands are phospholipase A_2 and venom nerve growth factor from *Naja* species.

Fraction	Accession	-10lgP	Coverage (%)	#Peptides	#Unique	Avg. Mass	Description	Origin of species
**P1**	**Q9I900**	**255.4**	**78**	**36**	**5**	**16097**	**Acidic phospholipase A_2 D**	***Naja sputatrix***
	**P00596**	**231.01**	**79**	**26**	**2**	**16271**	**Acidic phospholipase A_2 1**	***Naja kaouthia***
	**Q91133**	**216.42**	**79**	**20**	**2**	**15949**	**Acidic phospholipase A_2 2**	***Naja atra***
	**Q6T179**	**210**	**52**	**23**	**2**	**14198**	**Acidic phospholipase A_2 4 (Fragment)**	***Naja sagittifera***
	**P01140**	**198.37**	**91**	**12**	**1**	**13023**	**Venom nerve growth factor**	***Naja naja***
	**P60043**	**188.05**	**48**	**17**	**3**	**14003**	**Basic phospholipase A_2 1 (Fragment)**	***Naja sagittifera***
	**P25498**	**185.72**	**66**	**14**	**3**	**13229**	**Acidic phospholipase A_2 E**	***Naja oxiana***
	**P60044**	**174.18**	**50**	**15**	**1**	**14073**	**Acidic phospholipase A_2 2 (Fragment)**	***Naja sagittifera***
	**P60045**	**168.88**	**67**	**13**	**2**	**13969**	**Acidic phospholipase A_2 3 (Fragment)**	***Naja sagittifera***
	**Q92084**	**108.77**	**49**	**5**	**5**	**16189**	**Neutral phospholipase A_2 muscarinic inhibitor**	***Naja sputatrix***

Supplemental Table S1b: Protein profile of fraction P2 eluted from G75 Superdex™ column. Protein gel bands were tryptic digested and identified by ESI-LC-MSMS. Protein profile in P2 exhibited the presence of acidic and neutral phospholipase A2, venom nerve growth factors and thaicobrin.

Fraction	Accession	-10lgP	Coverage (%)	#Peptides	#Unique	Avg. Mass	Description	Origin of species
**P2**	**Q91133**	**231.54**	**63**	**23**	**1**	**15949**	**Acidic phospholipase A_2 2**	***Naja atra***
	**P01140**	**211.23**	**97**	**16**	**2**	**13023**	**Venom nerve growth factor**	***Naja naja***
	**P01140**	**200.75**	**95**	**12**	**11**	**13023**	**Venom nerve growth factor**	***Naja naja***
	**Q6T179**	**200.22**	**52**	**17**	**1**	**14198**	**Acidic phospholipase A_2 4 (Fragment)**	***Naja sagittifera***
	**A4FS04**	**192.21**	**76**	**13**	**2**	**13188**	**Acidic phospholipase A_2 natratoxin**	***Naja atra***
	**P25498**	**187.51**	**66**	**13**	**4**	**13229**	**Acidic phospholipase A_2 E**	***Naja oxiana***
	**P82885**	**184.38**	**69**	**10**	**5**	**12038**	**Thaicobrin**	***Naja kaouthia***
	**P60044**	**180.37**	**60**	**13**	**1**	**14073**	**Acidic phospholipase A_2 2 (Fragment)**	***Naja sagittifera***
	**Q92084**	**154.38**	**68**	**7**	**2**	**16189**	**Neutral phospholipase A_2 muscarinic inhibitor**	***Naja sputatrix***
	**P61898**	**149.37**	**94**	**10**	**1**	**13064**	**Venom nerve growth factor**	***Naja atra***

Supplementary 2

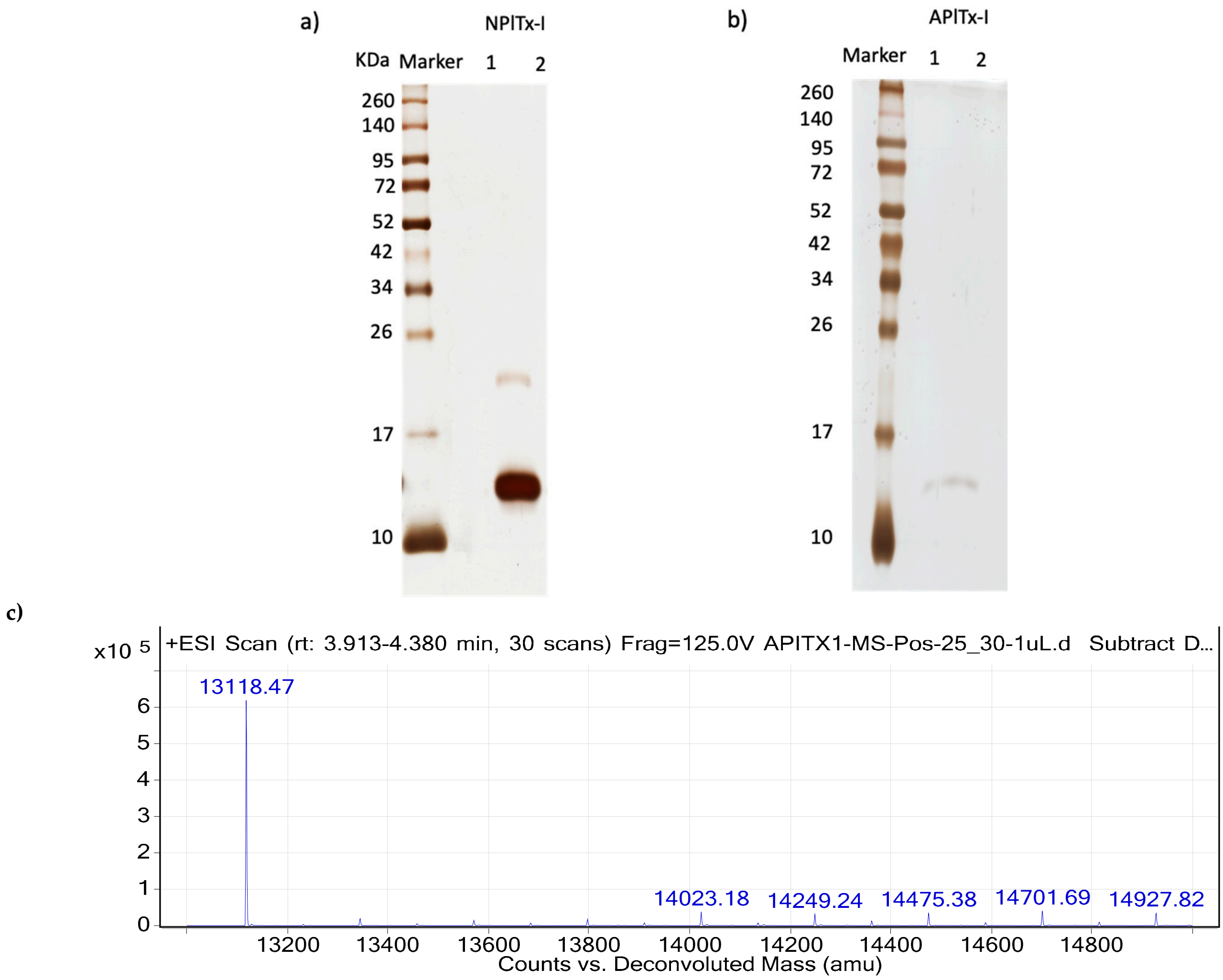

Supplementary Figure S2: Protein bands of two fractions collected from the C18 reverse phase column separated in SDS-PAGE gel and stained with silver staining. The first peak eluted from the C18 column named NPlTx-I were evaluated in Glycine SDS-PAGE both in nonreducing (lane 1) and reducing (lane 2) (a). NPlTx-I showed a dominant band at MW 13KDa and another low express band at 20KDa. The second elution named APlTx-I showed low expression of a protein band at a lower migration of 13KDa (b). Intact protein analysis using accurate mass LC-MS showed that APlTx-II has a molecular weight of 13,118.47 Da suggested different isomers or consisted of PLA2 fragment due to the mass differences of ~2KDa with A2-EPTX-Nsm1a, which been originated from NPlTx-I. All protein bands from NPlTx-I and APlTx-II were subjected to in-gel tryptic digestion, and protein identification was performed by ESI-LC-MSMS (Supplemental table 2a and 2b).

Supplemental Table S2a: Protein profile of fraction NPlTx-I eluted from C18 reverse-phase column. Protein gel bands were tryptic digested and identified by ESI-LC-MSMS. Protein profiles of NPlTx-I indicated similarity with phospholipase A_2 from *Naja* species.

Fraction	Accession	-10lgP	Coverage (%)	#Peptides	#Unique	Avg. Mass	Description	Origin of species
**NPlTx-I**	Q9I900	204.12	71	20	1	16097	Acidic phospholipase A_2 D	*Naja sputatrix*
	P00596	192.23	80	20	2	16271	Acidic phospholipase A_2 1	*Naja kaouthia*
	Q91133	184.5	63	14	1	15949	Acidic phospholipase A_2 2	*Naja atra*
	Q6T179	162.87	52	13	1	14198	Acidic phospholipase A_2 4 (Fragment)	*Naja sagittifera*
	P60043	149.35	38	10	1	14003	Basic phospholipase A_2 1 (Fragment)	*Naja sagittifera*
	P25498	146.69	54	10	2	13229	Acidic phospholipase A_2 E	*Naja oxiana*
	P60045	137.27	44	9	1	13969	Acidic phospholipase A_2 3 (Fragment)	*Naja sagittifera*
	P60044	133.62	50	8	1	14073	Acidic phospholipase A_2 2 (Fragment)	*Naja sagittifera*
	P00601	127.06	47	11	1	13360	Acidic phospholipase A_2 DE-III	*Naja melanoleuca*
	Q92084	118.58	68	8	3	16189	Neutral phospholipase A_2 muscarinic inhibitor	*Naja sputatrix*

Supplemental Table S2b: Protein profile of fraction APlTx-I eluted from C18 reverse-phase column. Protein gel bands were tryptic digested and identified by ESI-LC-MSMS. Protein profiles of APlTx-I indicated 49% similarity with neutral phospholipase A_2 originated from *Naja sputatrix.*

Fraction	Accession	-10lgP	Coverage (%)	#Peptides	#Unique	Avg. Mass	Description	Origin of species
**APlTx-I**	Q92084	95.93	49	5	0	16189	Neutral phospholipase A_2 muscarinic inhibitor	*Naja sputatrix*
	Q92085	95.6	49	5	1	16175	Neutral phospholipase A_2 B	*Naja sputatrix*

Supplementary 3

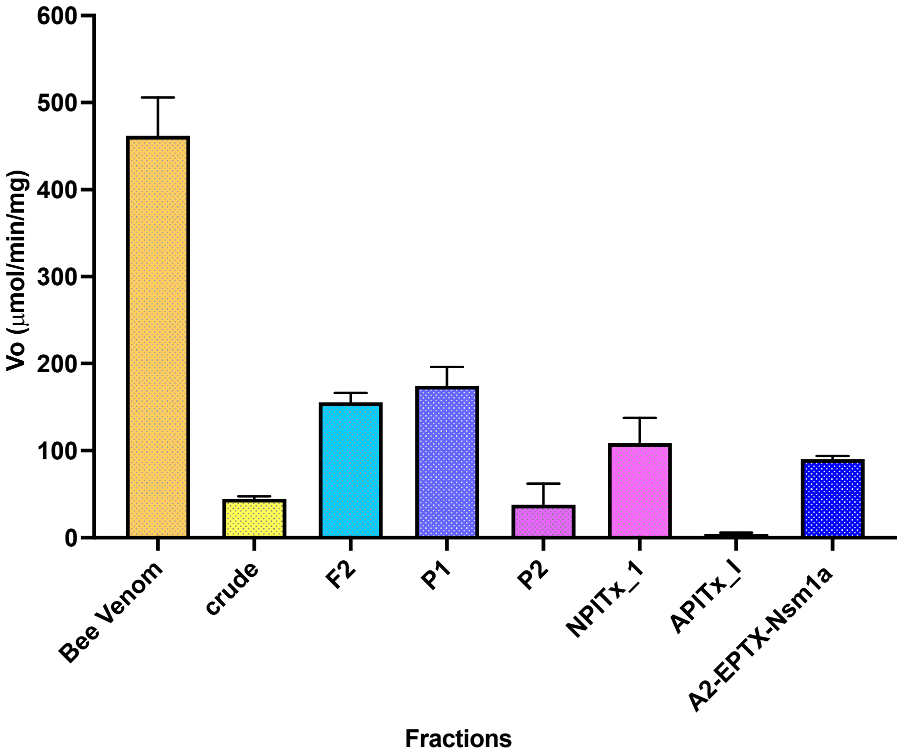

Supplementary Figure S3: Confirmation of PLA_2 activity in crude and its fractions that identified with the presence of phospholipase obtained from ESI-LC-MS/MS. A similar amount of each selected fraction and crude *N. sumatrana* venom (0.045 µg/ml) indicated hydrolysis of heptanoyl phosphatidylcholine, indicating the presence of secretory PLA_2. Fraction 2 (F2) isolated from G50 Sephadex™ column, fraction 1 (P1) eluted from G75 Superdex™ column, and semi-purified PLA_2, NPlTx-I isolated from reverse phase C18 column showed higher secretory PLA_2 activity as compared to fraction P2. This activity suggested due to other toxins such as venom nerve growth factor and thaicobrin (Supplemental table 1b), which probably more dominant in the P2. In addition, the activity of secretory PLA_2 in APlTx-I demolished at the same concentration. The assay was performed in triplicate, and bee venom was used as the positive control.
